# The axonemal structure of mouse respiratory cilia

**DOI:** 10.1186/2046-2530-1-S1-P55

**Published:** 2012-11-16

**Authors:** HU Ueno, TI Ishikawa, HB Bui, TY Yamaguchi

**Affiliations:** 1 Tohoku University, Japan; 2 Paul Scherrer Institute, Switzerland

## 

Mucociliary clearance on the surface of the tracheal lumen is an important component of lung defense against dust mites, viruses. Fluid on the surface of the tracheal lumen flows from the lungs to the oropharynx as a result of effective ciliary motion. However, the axonemal structure and the mechanisms by which discretely distributed ciliary cells generate directional flow have been unknown. In this study, we examined the motion of individual cilia with 7-9 nm spatial precision by labeling the tip of each cilium with quantum dots, and detected an asymmetric beating pattern in individual cilia. Then, in order to understand the molecular mechanisms of asymmetric ciliary motion, we analyzed the axonemal structure of respiratory cilia by cryo-electron tomography and image processing. Interestingly, isolated cilia from the mouse tracheal lumen have curvature in the non-active state, and the densities of two of the eight inner dynein arms—dyneins b/g and e, which have been described in *Chlamydomonas* flagella—were missing from at least two doublet microtubules in respiratory cilia. These results suggest that the asymmetric axonemal structure contributes to asymmetric ciliary motion. Lastly, we investigated the flow field generated by the asymmetric ciliary motion of sparsely distributed ciliated cells in mouse trachea. Although the flow directions generated by individual ciliated cells were unsteady, the velocity field was found to be directional. These results indicate that mouse respiratory cilia with the asymmetric axonemal structure on sparsely distributed ciliated cells can generate overall directional flow from the lungs to the oropharynx.

